# NLRC5: potential novel non-invasive biomarker for predicting and reflecting the progression of IgA nephritis

**DOI:** 10.1186/s12967-018-1694-1

**Published:** 2018-11-19

**Authors:** Yusa Chen, Huihui Li, Chenggen Xiao, Xiangli Zeng, Xiangcheng Xiao, Qiaoling Zhou, Ping Xiao

**Affiliations:** Department of Nephrology, Xiangya Hospital, Central South University, 87 Xiangya Road, Kaifu District, Changsha, 410008 Hunan China

**Keywords:** NLRC5, IgA nephritis, Renal biopsy

## Abstract

**Background:**

The nucleotide oligomerization domain-like receptor subfamily C5 (NLRC5) is primarily expressed in the adaptive and innate immune systems. NLRC5 was recently discovered to regulate immunity and inflammatory responses. Abnormal immune and inflammatory responses are considered critical pathogenesis in IgA nephritis (IgAN). However, the role of NLRC5 in IgAN is unknown. We previously showed that NLRC5 can be detected in patients with IgAN; herein, we further examined the pathophysiological significance of NLRC5 in the serum and renal deposits of patients with IgAN. This study is the first to find that NLRC5 is closely correlated with IgAN.

**Methods:**

IgAN patients (n = 50) who were diagnosed by renal biopsy provided blood and renal biopsy tissue, and age-matched healthy control subjects (blood donators n = 22; tissue donators n = 5) were included. Renal biopsies were diagnosed, and blood biochemical parameters were tested. Serum creatinine, urea, proteinuria, haematuria, albumin, and immunoglobulin A levels were recorded. Serum NLRC5 concentrations were detected by enzyme-linked immunosorbent assay, and tissue NLRC5 expression in kidney tissue was detected by immunohistochemical analysis. ROC curve analysis was used to evaluate the diagnostic value of the serum NLRC5 concentration in IgAN.

**Results:**

Serum NLRC5 concentration was significantly decreased in the IgAN group compared to that in the healthy control group (*P *< 0.0001), especially in S1 (Oxford classification) patients (*P *< 0.0001). Furthermore, serum NLRC5 concentration had a negative correlation with Lee’s grade (*r *= 0.3526, *P *= 0.0060) and proteinuria levels (*r *= 0.4571, *P *= 0.0004). Tissue NLRC5 expression was significantly increased in the IgAN group compared to that in the healthy control group (*P *< 0.0001); a more significant increase was identified in the S1 group (*P *< 0.05) and had a positive correlation with Lee’s grade (*r *= 0.497, *P *< 0.0001). We proposed a cut-off value of 1415 pg/ml for serum NLRC5 concentration, which was able to predict IgAN with 77.27% sensitivity and 87.5% specificity.

**Conclusions:**

Serum NLRC5 concentrations in IgAN are significantly decreased, and tissue NLRC5 expression is significantly increased in IgAN renal tissue, which is consistent with pathological severity. This finding suggests that NLRC5 could potentially be a diagnostic index and represents a prognostic factor in IgAN patients.

## Background

IgAN is the most common type of primary glomerulonephritis [1]. However, the pathogenesis of IgAN is not completely understood. IgAN appears to be a systemic disease in which the kidneys, innocent bystanders in this situation, are damaged because IgAN frequently recurs after transplantation [2]. Conversely, Iwata et al. [3]. reported a case of IgAN in which IgA deposition disappeared after peripheral blood stem cell transplantation for acute lymphocytic leukaemia.

The clinical manifestations of IgAN are highly variable, and the diagnostic hallmark of IgAN is the predominance of IgA deposits, either alone or with IgG, IgM, or both, in the glomerular mesangium. In 1982, Lee et al. [4]. proposed a grading system for IgAN, referred to as Lee’s grade, which considers histologic lesions that may predict the prognosis of IgAN and is easy to operate and apply. The Oxford classification was published in 2009, in which pathologists blinded to the clinical data scored renal biopsies from all IgAN patients for pathological variables identified as reproducible by an iterative process, and four variables were proposed, which were subsequently shown to have independent value in predicting renal outcomes [5]. The Lee’s grade and Oxford classification are both considered to predict clinical prognosis of IgAN, but both of them need renal biopsy. Furthermore, evaluation of IgAN severity and progression by repeated renal biopsy is not practical because it is an invasive technique.

Non-invasive screening and monitoring for IgAN is challenging because of different limitations. Activated complement C3 [6], increased serum levels of uric acid [7, 8] and plasma levels of fibroblast growth factor 23 [9] have been reported to be associated with severe pathological lesions, severe proteinuria, or a poor clinical outcome. However, these findings may not be unique to IgAN, as the sensitivity and specificity of these indicators are insufficient to replace the renal biopsy testing standard and they do not exhibit significant correlation with the progression of IgAN [6–9]. Thus, the investigation of novel non-invasive biomarkers for evaluation of the diagnosis, severity and progression of IgAN has been the focus of research.

The proteins of nucleotide binding oligomerization domain-like receptors (NLRs) are a family of intracellular proteins distributed in the cytoplasm and nucleus that play an important role in inflammation and immunity [10, 11]. NLRC5 (also known as NOD4, NOD27 or CLR16.1) consists of 1866 aa with 27 C-terminal leucine-rich repeats (LRRs) and is the largest of the members of the NLR family, which have recently gained increased attention as important components of both the immune and inflammatory response systems [12, 13]. As a critical regulator of immune responses, NLRC5 is an important target for modulating innate immune signalling and regulation and plays critical roles in MHC class I expression, innate immune signalling and antiviral innate immune responses [14]. Lupfer et al. [15]. found that Nlrc5-deficient mice show decreased MHC-I expression on haematopoietic cells, fewer CD8^+^ T cells prior to infection, and impaired effector function. Li et al. [16]. found that NLRC5 deficiency reduced the activation of CD4^+^ T cells, which are important mediators after renal ischaemia reperfusion injury. Regarding immune-related nephritis—IgAN, we hypothesized that the level of NLRC5 in IgAN patients may be different from that in healthy people and that an evaluation of the serum concentration and tissue expression of NLRC5 may provide a reliable non-invasive screening method for IgAN diagnosis and prognosis. Furthermore, finding a new pathogenesis for IgAN was a goal of this study. In our study, we examined the serum NLRC5 concentration to investigate whether non-invasive serum NLRC5 measurement was closely correlated with other indexes, such as haematuria, proteinuria, and uric acid, and the severity of renal pathology in IgAN patients and then calculated tissue NLRC5 expression to inquire into whether it was correlated with the severity of renal pathology or other indexes. The goals of this study were to establish a correlation between changes in NLRC5 and the severity of IgAN and then to reveal the diagnostic and prognostic values of NLRC5 in evaluating IgAN.

## Materials and methods

### Study subjects

This prospective study was conducted from December 2016 to February 2018, and it was approved by the medical ethics committee of Xiangya Hospital, Central South University (Ethical Code: 201612675). Furthermore, it was carried out in accordance with the ethical principles of the Helsinki Declaration. Written informed consent was received from all subjects before enrolment.

We included patients with a renal biopsy diagnosis of IgAN (n = 50) and healthy controls (serum controls: n = 22; biopsy controls: n = 5) who were matched for age and gender. Patient inclusion criteria included a percutaneous renal biopsy diagnosis of IgAN and age < 60 years old. Patient exclusion criteria included secondary glomerulopathy caused by tumour or other diseases; tubulointerstitial lesions caused by other factors such as drugs and metabolism; and tumour or other diseases that could cause NLRC5 alterations. Healthy serum controls were free of the conditions stated in the exclusion criteria and were recruited during physical check-ups, while healthy biopsy controls were recruited from normal renal tissue in patients with suprarenal epithelioma.

## Methods

Baseline information, including race, age, gender, history of health and medication use, was obtained at admission.

Ten millilitres of blood was drawn into tubes, which were centrifuged (3000 rpm at room temperature for 15 min), and the serum was immediately aliquoted and stored at − 80 °C until the assay was performed. Measurements of biochemical parameters, such as creatinine, urea, proteinuria, haematuria, serum albumin, uric acid and serum immunoglobulin A, were performed in a clinical laboratory at the hospital. All samples were thawed only once, immediately before use.

Serum levels of NLRC5 were measured using commercially available ELISA kits (Wuhan Abebio Science Co. Ltd., China; Catalogue #AE30903HU). According to the instructions, the intra-assay and interassay coefficients of variation for NLRC5 were < 8% and < 12%, respectively.

IgAN renal biopsy specimens consistent with the serum of patient donors were obtained. Healthy control biopsy samples were collected from normal renal tissue at a considerable distance from the cancer tissue in patients with suprarenal epithelioma. Diagnosis of the renal biopsies was provided by two pathologists at the same time in our hospital. Lee’s grade and the Oxford classification method were used to grade the IgAN pathology. Lee’s grade: Grade I: Most of the glomeruli are normal, occasionally mild mesangial enlargement (segmental) with or without cell proliferation, and no change in the renal tubules and interstitium; Grade II: The glomerulus shows focal mesangial proliferation and sclerosis (< 50%) with rare small crescents and no damage to renal tubules and interstitium; Grade III: glomerular diffuse mesangial proliferation and enlargement (occasionally focal segments) with occasional crescents; renal tubules and interstitial changes with focal renal interstitial oedema, occasional cell infiltration, and rare tubular atrophy; Grade IV: Severe diffuse mesangial hyperplasia and sclerosis, partial or total glomerulosclerosis, and crescent bodies (< 45%) were seen in glomerulopathy, renal tubular atrophy, interstitial infiltration of kidney, occasionally renal interstitial foam cells; Grade V: The nature of glomerular lesions is similar to Grade IV, but more serious, glomerular crescent formation > 45%; tubular and renal interstitial lesions are similar to Grade IV, but more serious (we had no patients in grade V). Oxford classification: S0 was defined as the absence of segmental glomerulosclerosis, but S1 was defined as its presence; M0 was defined as a mesangial hypercellularity score less than 0.5 and M1 was a score higher than that; E0 was defined as the absence of endocapillary hypercellularity, but E1 was defined as its presence; T0 was defined as tubular atrophy/interstitial fibrosis less than 26% of the cortical area; T1 was defined as tubular atrophy/interstitial fibrosis within 26–50% of the cortical area, and T2 was defined as tubular atrophy/interstitial fibrosis in greater than 50% of the cortical area.

For immunohistochemistry, the renal tissue was embedded in paraffin, and the paraffinized renal tissue sections (2 µm) were stained by a Peroxidase Labelled Streptavidin/Peroxidase staining kit (Zhongshan Golden Bridge, Beijing, China, SP-9000). Paraffinized sections were routinely dewaxed, incubated in 3% H_2_O_2_ in deionized water for 5–10 min, rinsed with distilled water, and soaked in phosphate-buffered solution (PBS) for 5 min. They were then incubated in citrate buffer (pH = 6.0) for 20 min at 80 °C, rinsed with PBS 3 times for 3 min each time and covered with reagent A (blue liquid) to incubate for 10–15 min at room temperature. Afterwards, a monoclonal NLRC5 antibody was added (Abcam, USA; ab105411, anti-rabbit, and 1:100) for incubation at 4 °C overnight. The sections were rinsed 3 times for 3 min in PBS, reagent B (yellow liquid) was added, and the sections were incubated at room temperature for 10 min, followed by rinsing in PBS 3 times for 3 min. Reagent C (orange liquid) was then added to the sections, which were incubated at room temperature for 10 min, followed by 3 rinses in PBS as described above. The sections were then dyed with a chromogenic agent for 10 min, rinsed with sufficient tap water to terminate the dyeing reaction, counterstained with haematoxylin, washed with tap water until they were blue, dehydrated, and finally sealed with neutral balsam. The samples were examined using an H-7700 transmission electron microscope (Hitachi, Japan). Semiquantitative calculation of tissue NLRC5 expression was performed with Image-Pro plus 6.0 immunohistochemical analysis software. The average optical density (AOD) quantitative analysis index was used to describe tissue NLRC5 expression.

### Statistical analyses

Continuous variables with a normal distribution were expressed as the mean ± standard (M ± SD) or as appropriate, unless otherwise indicated. Variables with a skewed distribution were expressed as the median (interquartile range). Nominal variables were expressed as a count or as a percentage. Data analyses were performed using Statistical Package for the Social Sciences, version 25.0 (SPSS Inc, Chicago IL, USA). The Chi square test was used to statistically analyse numeration data. Student’s t-test was used to statistically analyse comparisons between two groups, and one-way analysis of variance (ANOVA) was used for analysis among three or more groups. We performed logistic regression analysis and Pearson’s or Spearman’s correlation tests to specify relationships among the variables, as indicated in the figure legends. We used ROC curve analysis to evaluate the diagnostic value and to define the diagnostic cut-off value of serum NLRC5 concentrations in IgAN. Additionally, ROC curve analysis was used to determine the range of suspicious values. *P *< 0.05 indicated a significant difference, and *P *< 0.0001 indicated a highly significant difference.

## Results

The background state of the participants was well controlled.

The study included serum samples from 50 patients with IgAN, 22 age- and sex-matched healthy serum controls and 5 age- and sex-matched tissue controls. The renal biopsy samples of 50 patients (the same individuals who donated the serum samples) with IgAN were also included in the study. All participants were of Han ethnicity. The baseline characteristics for all subjects are shown in Table 1. No significant differences were found between the IgAN patients and controls with regard to age, gender, or levels of creatinine, serum albumin, uric acid and IgA (Table 1), indicating that both groups of participants were comparable to each other. In our study, we found that proteinuria was different between IgAN patients and controls because IgAN patients usually present with proteinuria [17]. However, we found that only haematuria was different between the IgAN patients and serum controls, there were no differences between IgAN patients and tissue controls because the patients with suprarenal epithelioma also had haematuria [18].

**Table 1:** Baseline characteristics of the study population characteristic

	IgAN	Control		P-value
	(n = 50)	(Serum: n = 22)	(Tissue: n = 5)	(Serum/tissue)
Age (years)	32.90 ± 1.36	34.04 ± 1.98	41.40 ± 4.64	0.6353/0.0591
Female (%)	38 (64)	12 (54.5)	3 (60)	0.3953/0.1774
Creatinine (µmol/l)	79.91 ± 2.39	73.65 ± 3.39	85.33 ± 6.65	0.1397/0.4558
Urea (mmol/l)	4.54 ± 0.19	4.01 ± 0.27	4.57 ± 0.40	0.1219/0.9590
Proteinuria (g/day)	0.85 ± 0.14	0.10 ± 0.01	0.21 ± 0.01	0.0005*/0.0046*
Haematuria (/ml)	203.30 ± 54.29	0.61 ± 0.21	287 ± 0.21	0.013*/0.32
Albumin (g/l)	38.45 ± 0.69	38.07 ± 0.96	36.17 ± 0.55	0.7495/0.5326
IgA (mg/l)	2910 ± 159.92	2924 ± 219.61	3224 ± 168.93	0.9595/0.6427
Uric acid (mmol/l)	352.71 ± 13.34	350.12 ± 15.87	376.1 1 ± 10.54	0.9089/0.7842

Continuous variables were expressed as M ± SD. Categorical values were presented as frequencies (percentages). By *Unpaired t test*. *P < 0.05

All of the renal biopsy specimens were classified by Lee’s grade; however, only 40 specimens had been typed using Oxford classification as presented in Table 2. The specimens were classified according to Lee’s grade as follows: grade I: n = 18; grade II: n = 25; grade III-IV: n = 7. They were grouped into the following Oxford classification categories: S0: n = 27; S1: n = 13; M0: n = 39; M1: n = 1; E0: n = 3; E1: n = 37; T0: n = 39; T1; n = 1.

**Table 2:** Basic pathological classification of IgAN

Pathological types	IgAN
Lee’s grade	(n = 50)
I	18
II	25
III–IV	7
Oxford classification	(n = 40)
S0/S1	27/13
M0/M1	39/1
E0/E1	3/37
T0/T1	39/1

I–IV, Lee’s grade I–IV; M, mesangial hypercellularity score; S, segmental glomerulosclerosis; E, endocapillary hypercellularity; T, tubular atrophy/interstitial fibrosis

### Serum NLRC5 concentrations were negatively correlated with Lee’s grade and were significantly lower in S1 IgAN patients than in S0 patients

ELISA was used to evaluate whether serum NLRC5 concentrations were different between patients with IgAN and controls. Subsequently, we evaluated whether serum NLRC5 concentrations were different among IgAN patients grouped by Lee’s grade or the Oxford classification.

**Fig. 1:**
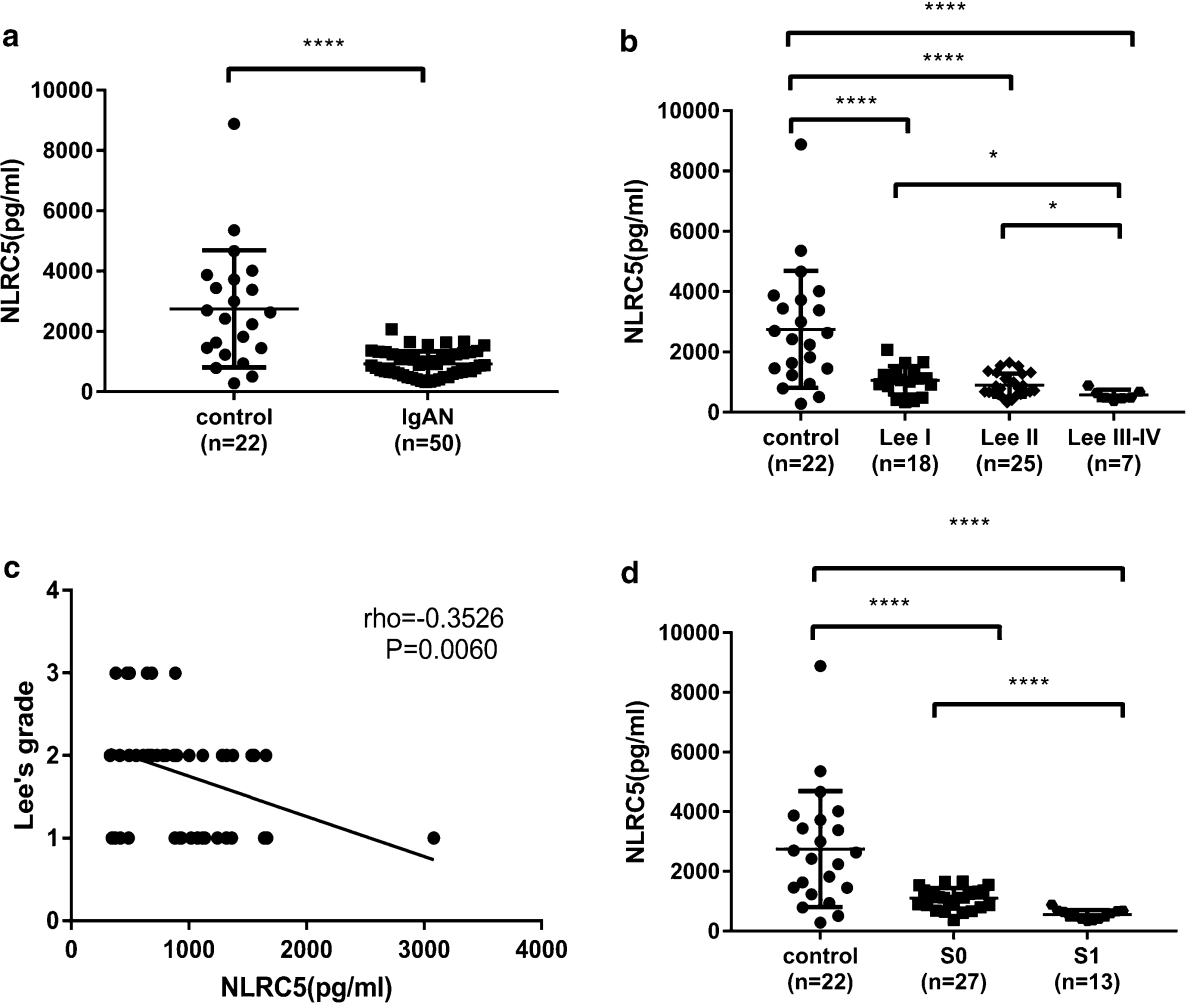
Serum NLRC5 concentrations were reduced in IgAN patients, had a negatively correlation with Lee’s grade. **a** The Serum NLRC5 concentration in IgAN patients and control subjects (tested); **b** The concentration of serum NLRC5 in Lee’s grade I-IV IgAN patients; **c** The correlation analysis between serum NLRC5 concentration and Lee’s grade; **d** Serum NLRC5 concentrations in controls, S1 and S0 of the Oxford classification. **a** by *Unpaired t test*; **b** and **d** by *Ordinary one*-*way ANOVA test*; **c** by *Pearson test*.*P < 0.05, ****P < 0.0001

Because the sample size was too small, 7 cases of Grade III and Grade IV could not be effectively analysed, so they were combined into one group. There were no grade V samples in our collection of specimens, so we had Lee’s grade I-IV patients, as presented in Table 2. The serum NLRC5 concentrations were significantly lower in the IgAN patients than in the controls (Fig. 1a, 931.9 ± 59.06 pg/ml vs 2754 ± 414.1 pg/ml; *P *< 0.0001). Furthermore, the serum NLRC5 concentrations were lowest in Lee’s grade III-IV patients (Fig. 1b, *P *< 0.0001). We found that they were negatively correlated with Lee’s grade (Fig. 1c, r = − 0.3526; *P *= 0.0060).

The Lee’s grade system considers renal interstitial injury to be associated with glomerular lesions [19], but no renal interstitial fibrosis was used as an independent indicator for predicting renal outcome. In clinical work, most patients are concentrated at the III level, and the difference between patients is poor. Hence, the relationships between the IgAN Oxford classification and serum NLRC5 concentrations were studied. Our research found that there was a difference between S0 and S1 in IgAN patients with regard to serum NLRC5 concentrations, which was marked by a decrease in S1 patients (Fig. 1d, *P *< 0.0001). This finding may suggest that serum NLRC5 concentrations are a good predictor of IgAN prognosis.

### Serum NLRC5 concentration was negatively correlated with proteinuria measured by biochemical detection in IgAN patients

**Fig. 2:**
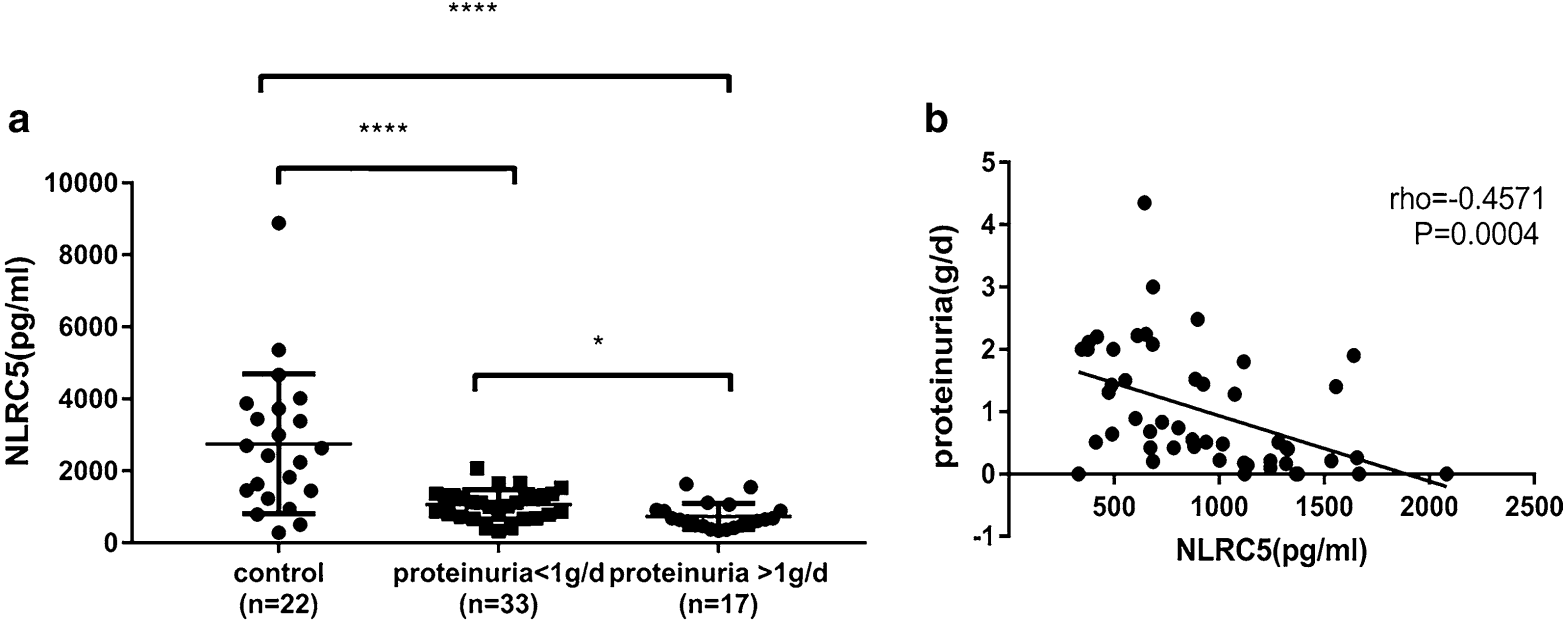
Serum NLRC5 concentrations negatively correlated with the proteinuria of IgAN patients. **a** Serum NLRC5 concentration in IgAN patients with different concentration of proteinuria (more than 1 g/day and less than 1 g/day); **b** Correlation analysis between serum NLRC5 concentration and proteinuria concentration. **a** by *Ordinary one*-*way ANOVA*; **b** by *Pearson* test. *P < 0.05, ****P < 0.0001

In IgAN, the clinical indicators of prognosis have been basically agreed upon: if the proteinuria concentration is more than 1 g/day, the patients tend to have a poor outcome [20, 21]. Our study found that the serum NLRC5 concentration showed obvious differences in IgAN patients with regard to proteinuria at concentrations of more than 1 g/day and less than 1 g/day (Fig. 2a, *P *< 0.05). Furthermore, we evaluated whether serum NLRC5 concentrations were correlated with proteinuria and other parameters measured using biochemical detection methods in IgAN patients, and we found that serum NLRC5 concentration had a significant negative correlation with proteinuria concentration in IgAN patients (Fig. 2b, *r *= − 0.4571; *P *= 0.0004).

The relationships between other parameters and serum NLRC5 concentrations were also studied, although we did not find any correlations between serum NLRC5 concentrations and these parameters (*P *> 0.05, Table 3).

**Table 3:** Correlations between serum NLRC5 concentration and blood biochemical parameters in IgAN patients

	NLRC5	
	*r*	*P*
Proteinuria	− 0.4571	0.0004*
Urea (mmol/l)	0.0459	0.3757
Haematuria (/ml)	0.1230	0.3996
Albumin (g/l)	0.0926	0.2611
IgA (mg/l)	− 0.2274	0.0561
Uric acid (mmol/l)	− 0.0153	0.4581

The correlations analysis between serum NLRC5 concentration and blood biochemical parameters in IgAN patients. Concentration were negatively correlated with proteinuria (*r *= − 0.4571, *P *= 0.0004) in IgAN patients. No significant correlations were found among NLRC5 and urea, haematuria, albumin, IgA and uric acid. By *Pearson test*. *P < 0.05

### Tissue NLRC5 expression in renal tissues was increased significantly in IgAN patients, positively correlated with Lee’s grade, and markedly increased in S1 IgAN patients compared with S0 patients

**Fig. 3:**
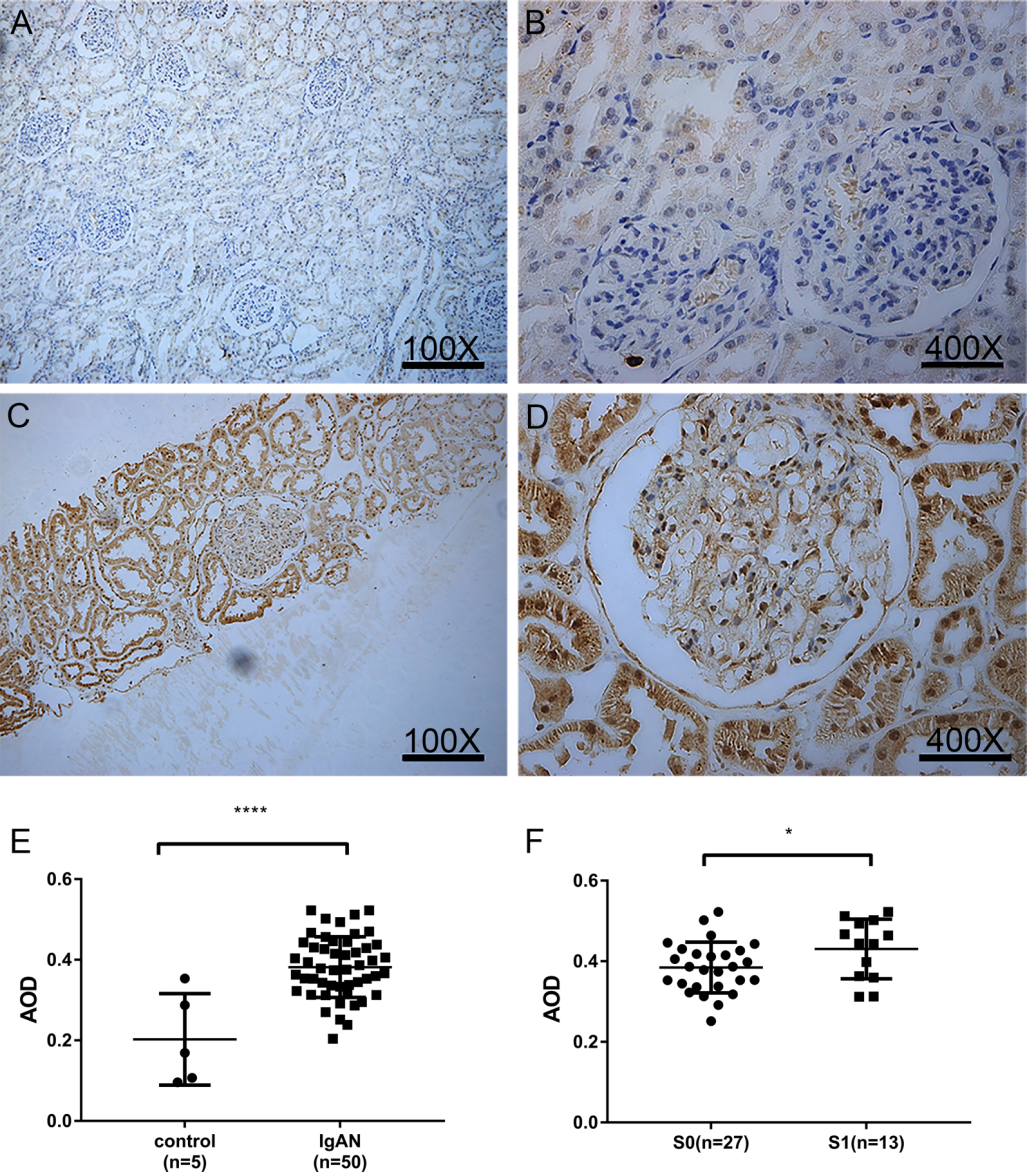
Tissue NLRC5 expression was different between IgAN patients and controls. Expression of NLRC5 in renal tissue of controls (**A**, **B**) and IgAN patients (**C**, **D**) by immunohistochemistry (**A**, **C** 100×), (**B**, **D**, 400×). **E** Relative quantitative analysis of renal tissue NLRC5 expression in controls and IgAN patients, the average optical density (AOD) was calculated by Image-pro Plus 6.0 immunohistochemical analysis software. **F** Quantitative analysis of renal tissue NLRC5 expression in S0 and S1 groups of IgAN patients according to Oxford classification. **E**, **F** by *Unpaired t test*. *P < 0.05, ****P < 0.0001

Because the diagnosis of IgAN depends on the immunohistopathological examination of renal tissue, we used immunohistochemical analysis to evaluate whether the expression of NLRC5 in the kidney was different between the IgAN patients and controls. The AOD quantitative analysis index was used to quantify deposited NLRC5. In normal kidney tissues, immunohistochemistry staining showed that NLRC5 expression was mainly in the nucleus of renal tubular epithelial cells, and little or no expression was shown in the glomerulus in control renal tissues. However, we found that NLRC5 expression was significantly increased in the renal specimens from IgAN patients, not only in the renal tubule but also in the glomerulus. Furthermore, the increased NLRC5 expression was primarily in the glomerulus, and it was mainly expressed in the cytoplasm and nucleus of mesangial cells and capillary endothelial cells in IgAN patients (Fig. 3). The main lesion of IgAN is glomerulus. Therefore, we believe that NLRC5 is closely related to the pathogenesis of IgAN. We calculated the AOD, and compared with the normal group, the IgAN group tissue NLRC5 expression showed a clear difference (Fig. 3E, 0.3819 ± 0.01066 vs. 0.2029 ± 0.05076, *P *< 0.0001), between S0 and S1 IgAN patients, a marked increase in S1 patients (Fig. 3F, *P *< 0.05).

### Tissue NLRC5 expression was not correlated with proteinuria measured by biochemical detection in IgAN patients or serum NLRC5 concentrations of IgAN patients

**Fig. 4:**
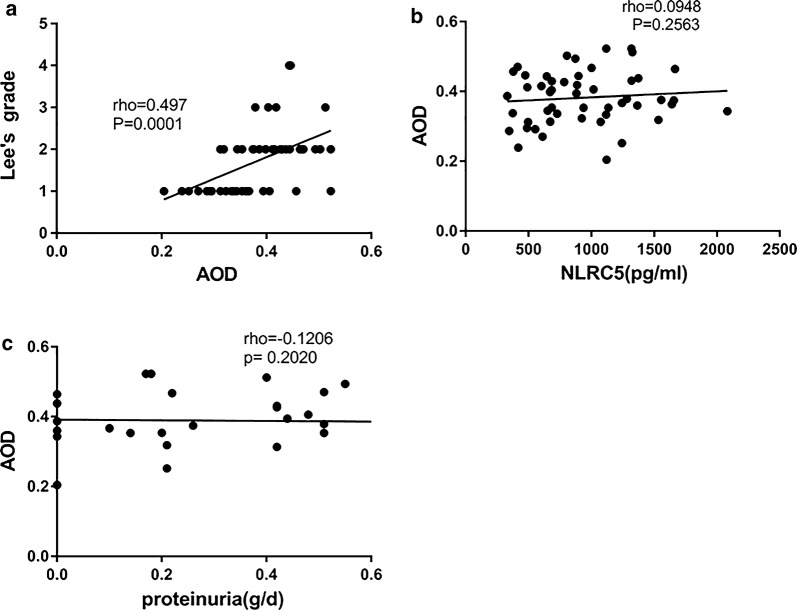
Correlations between Tissue NLRC5 expression and Lee’s grade, serum NLRC5 concentration, proteinuria in IgAN patients. Image-pro Plus 6.0 immunohistochemical analysis software was used to calculate the average optical density (AOD). **a** The correlation analysis between tissue NLRC5 expression (AOD) and Lee’s grade (*r *= 0.497, *P *= 0.0001); **b** The correlation analysis between tissue NLRC5 expression (AOD) and serum NLRC5 concentration (*r *= 0.0948, *P *= 0.2563); **c** The correlation analysis between tissue NLRC5 expression (AOD) and proteinuria concentration (*r *= 0.1206, *P *= 0.2020). By *Pearson test*. *P < 0.05, ****P < 0.0001

As demonstrated by the data, tissue NLRC5 expression had a positively correlation with Lee’s grade in the renal tissues of IgAN patients (Fig. 4a, *r *= 0.497, *P *= 0.0001). However, the tissue expression of NLRC5 had no significant correlation with serum NLRC5 concentrations or proteinuria concentration in IgAN patients (Fig. 4b, *r* = 0.0948, *P *= 0.2563; 4C *r *= 0.1206, *P *= 0.2020).

We proposed a cut-off value of 1415 pg/mL for serum NLRC5 concentration to distinguish IgAN patients from healthy controls.

**Fig. 5:**
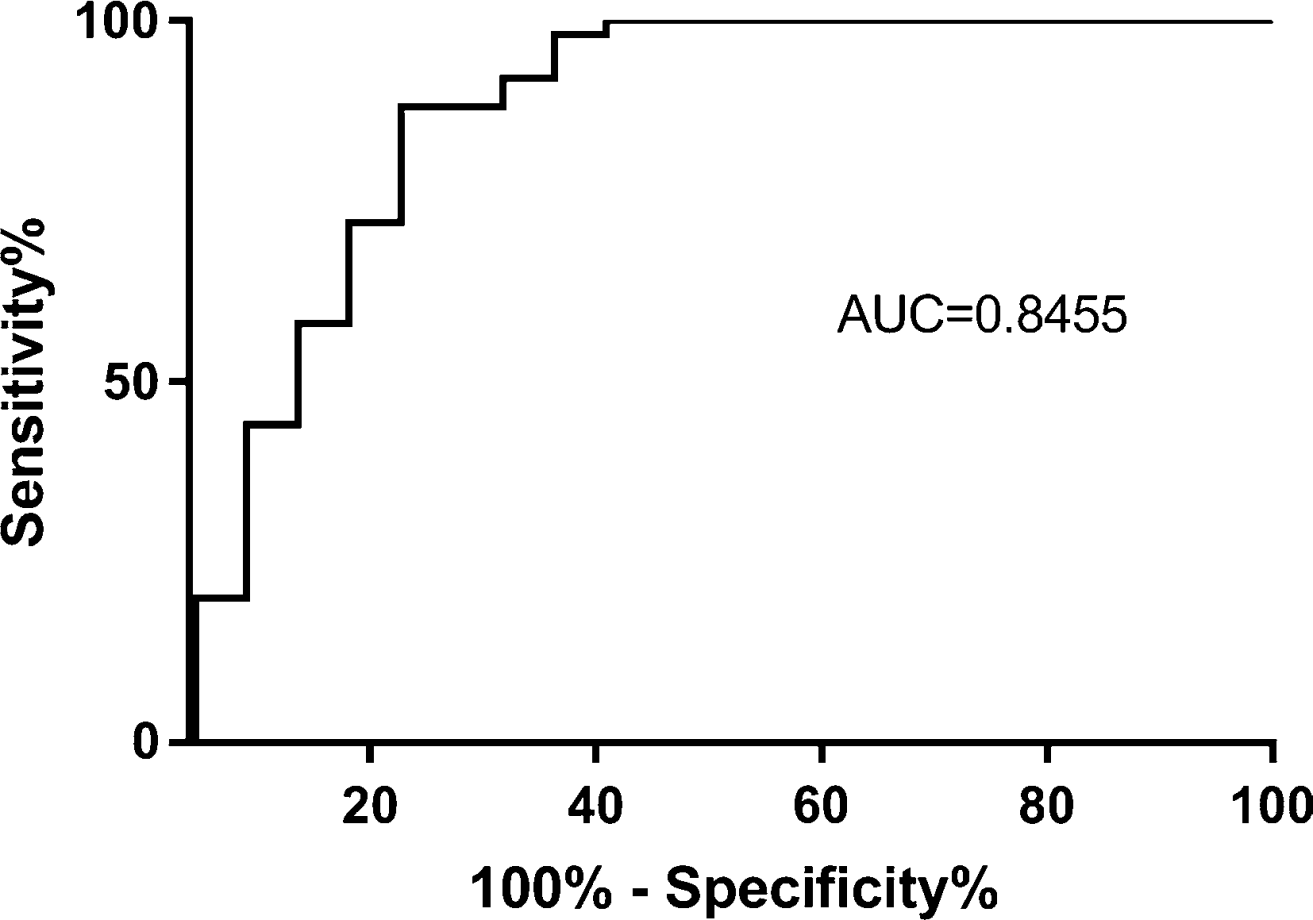
The cut-off value of serum NLRC5 concentration was 1415 pg/ml. The cut-off value of serum NLRC5 concentration was 1415 pg/ml to distinguish IgAN patients from the area under the curve was 0.8445 (95% CI 0.725–0.9659), and 1415 ng/ml was the maximum cut-off value. At this point, the PH had 77.27% sensitivity and 87.5% specificity

We discovered differences in serum NLRC5 concentrations between IgAN and healthy control subjects. Therefore, we intended to find the discrimination point to help diagnose IgAN. We proposed a cut-off value of 1415 pg/ml for serum NLRC5 concentration, which was able to predict IgAN with 77.27% sensitivity and 87.5% specificity. The area under the curve was 0.8455 (95% CI 0.725–0.9659), as shown in Fig. 5. We found that the IgAN patients who had almost normal NLRC5 levels were classified as Lee’s grade I-II, or to put it another way, the serum NLRC5 concentration was mildly decreased in patients with less serious pathology.

## Discussion

As one of the most common types of primary glomerulonephritis worldwide, IgAN is a chronic progressive renal disease with a high incidence of disease [1]. IgAN appears to be a systemic disease, and the kidneys are innocent bystanders in this situation [2]. Even today, the definitive diagnosis of IgAN still requires a renal biopsy.

In IgAN patients, we first found that the serum NLRC5 concentrations were significantly decreased, and tissue NLRC5 expression was significantly increased in IgAN renal tissue, which was correlated with pathological severity. No research is available regarding NLRC5 in IgAN, and we have not found a clear mechanism for NLRC5 in the pathogenesis of IgAN. However, we found that the phenomenon of NLRC5 in IgAN is similar to C3 complement in kidney diseases such as lupus nephritis, in which it was reported that C3 is associated with the activity and deterioration of lupus nephritis. Patients with low levels of serum C3 are thought to have active renal disease, and the serum C3 level represents the severity of the disease [22]; however, most patients were positive for complement C3 deposition in the kidney [23].

Innate immunity as a driving force in the pathogenesis of many kidney diseases (including IgAN) and involves a range of immunomodulatory mechanisms [24]. NLRC5, consistent with its role in immune regulation and host defence, is expressed in a wide spectrum of cell types and tissues but shows high abundance in immune-related cells and organs with mucosal surfaces [25]. Low serum levels of NLRC5 in IgAN patients may indicate abnormal immune regulation and host defence. The role of NLRC5 as an MHC class I transactivator seems to be greatest for T cells, B cells, and NK cells, but NLRC5 also plays some role in macrophages and DCs [26]. Some scholars have found that NLRC5 expression in T cells is required to protect them from NK cell-mediated elimination upon inflammation. NK cells surprisingly break tolerance even towards ‘self’ Nlrc5-deficient T cells under inflammatory conditions. In mice with chronic LCMV infection, the total CD8^+^ T-cell population is severely decreased, a phenotype reversed by NK cell depletion [27]. NLRC5 expression was significantly associated with the activation of CD8^+^ cytotoxic T cells and patient survival in multiple cancer types, the low expression of NLRC5 is correlated with the poor survival rate of cancer patients [28]. Moreover, IgAN is always exacerbated after an upper respiratory tract infection with haemolytic streptococcus [29]. Although we did not identify the cause of the reduction in serum NLRC5 in IgAN patients, we hypothesized that low expression of serum NLRC5 in IgAN patients, possibly similar to the manner in which NK cells break tolerance towards ‘self’ Nlrc5-deficient T cells under inflammatory conditions, may induce an abnormal immune response, initiating IgA1-circulating immune complex formation, deposition in the glomerular mesangium, and inflammatory cell infiltration to cause local kidney inflammation when patients are infected with haemolytic streptococcus. We hypothesized that lower NLRC5 levels could induce more severely abnormal immune responses to cause more serious disease in IgAN patients. This reason may explain why serum NLRC5 is negatively correlated with more advanced disease. Of course, further research is needed to confirm this hypothesis.

Iwata et al. [3] reported a case in which IgA deposition in an IgAN patient disappeared after peripheral blood stem cell transplantation for acute lymphocytic leukaemia, suggesting that some circulating factors may play an important role in IgAN and that IgAN appears to be a systemic disease in which the kidneys are damaged as innocent bystanders. When systemic diseases occur, abnormal substances (immune complexes) produced in the serum are deposited in the kidneys during filtration through the kidneys and cause kidney disease. Therefore, in many kidney diseases (including IgAN), some biomarkers are downregulated in serum and deposited or significantly expressed in the kidneys [30, 31]. We found that NLRC5 expression tendencies differed in serum and renal tissue between IgAN patients and controls, surprisingly, no correlation was found between the expression of NLRC5 in serum and tissue. However, there are studies that have shown a similar phenomenon in lupus nephritis. The levels of serum C1q were significantly lower in lupus nephritis patients than in normal controls, but serum levels of C1q were not associated with glomerular or vascular deposition of C1q [31]. Some research suggests that the variation in C1q in lupus nephritis may occur because serum C1q may contribute little to the deposition of C1q in the kidneys, while local production of C1q by endothelial cells [32], dendritic cells, and macrophages in the kidneys might be the main source of the local C1q deposition [33]. IgAN appears to be a systemic disease, with the kidney is an important target organ for immune complex deposits, and we found NLRC5 in IgAN like C1q complement in lupus nephritis. We assume that low expression of serum NLRC5 may contribute little to the deposition of NLRC5 in the kidneys but may serve as a trigger for inflammatory reaction in IgAN, local production of NLRC5 by endothelial cells, dendritic cells, and macrophages in the kidneys might be the main source of local NLRC5 deposition because of the local inflammatory response in IgAN, as NLRC5 promotes proinflammatory responses in parenchymal cells in acute renal failure (AKI) [16] and in mesangial cells in diabetic nephropathy (DN) [34], beyond the regulation of MHC class I genes. Therefore, we think that the lack of a correlation between the expression of NLRC5 in serum and tissue may be caused by the complex and diverse role of NLRC5 in different cells in IgAN.

With Lee’s grade, patients with grade II are considered benign, while patients with grade III to V have a tendency to have worsening renal function [4]. A meta-analysis [35] indicated that M, S, T and C were strongly associated with prognosis using the Oxford classification. Proteinuria concentration is one of the strongest predictors of outcome in IgAN, and the risk for renal failure increases with higher levels of proteinuria [20, 21]. Hence, most research studies use a proteinuria concentration cut-off of 1 g/day. From our data, we also surprisingly discovered that tissue NLRC5 expression was positively correlated with the Lee’s grade pathology category and was higher in group S1 than in group S0, thus suggesting that NLRC5 may potentially be an indicator for predicting disease outcomes. In our study, we discovered that serum NLRC5 concentrations were further decreased in the group with proteinuria concentrations > 1 g/day, and that the serum levels of NLRC5 were significantly correlated with proteinuria concentration. The correlation analysis for NLRC5 indicates that NLRC5 is potentially an important index that reflects IgAN prognosis. Furthermore, by immunohistochemistry staining, we found that the tissue NLRC5 expression was increased beyond the renal interstitium, and increased expression appeared in glomerular cells, mesangial cells and capillary endothelial cells, which was consistent with the pathological severity in IgAN patients. This phenomenon suggests that NLRC5 not only is involved in renal interstitial lesions but also acts as a vital mediator in glomerular mesangial cell proliferation and mesangial hyperplasia in the pathological process of renal glomeruli. We provided data from a single time point, but IgAN is a chronic kidney disease. Hence, additional studies addressing the temporal courses of NLRC5 values and their possible correlation with the development of IgAN disease are necessary. Our further studies will focus on glomerular cells which have significantly different NLRC5 expression between IgAN patients and controls.

We found no correlation between tissue NLRC5 expression and proteinuria, possibly because the number of samples was too small for accurate analysis or because proteinuria is mainly related to glomerular injury and the AOD calculates the degree of NLRC5 staining in renal intrinsic cells for the entire immunohistochemical image view, which includes the glomerulus and interstitium. Thus, comparisons between tissue NLRC5 expression and proteinuria concentration should not be made.

Until recently, several reports showed that NLRC5 has roles in other cells to induce inflammation. In hepatic fibrosis, NLRC5 is highly expressed in fibrotic liver tissue, and decreased expression was observed in the reversal stage through the NF-kB signalling pathway [36]; NLRC5 is also highly expressed in human keloid-a fibrotic tumours characterized by extensive ECM deposition and hyperproliferation of fibroblasts in the skin, and NLRC5 knockdown suppressed cell proliferation and ECM deposition involving transforming growth factor beta 1 (TGF-β1) and Smad2/3 [37]. In renal tissue, Li et al. [16]. found that gene silencing of NLRC5 decreased hypoxia-induced production of proinflammatory mediators and apoptosis in proximal tubule epithelial cells. Luan et al. [34]. found that NLRC5 promotes inflammation and fibrosis partly through effects on NF-kB and TGF-β/Smad pathways during DN progression. We found that NLRC5 expression differed in serum and renal tissue between IgAN patients and controls, suggesting that we may need to study the different roles of NLRC5 in immune and the local kidney inflammatory response in IgAN. We will investigate the specific mechanism underlying NLRC5 in IgAN in future studies.

Due to the small sample size, there were no significant differences detected between NLRC5 and other indexes, such as haematuria or uric acid, which were independent risk factors for poor prognosis of IgAN. The ROC curve analysis indicated that serum NLRC5 appeared to have a high sensitivity and specificity at a concentration of 1415 pg/ml. Taken together, the results of the present study present evidence that the pathological severity and poor prognosis in IgAN were associated with decreased serum NLRC5 concentrations and increased tissue NLRC5 expression. We provide evidence for the complex and diverse role of NLRC5 in human IgAN and suggest its potential application as a new biomarker in clinical detection of IgAN. This study provides a rationale for further clinical diagnosis and treatment after renal biopsy and for performing additional experimental studies investigating NLRC5 in the pathogenesis of IgAN. Although there is no substitute for renal biopsy for the diagnosis of IgAN, NLRC5 could possibly indicate the severity and prognosis of disease through non-invasive methods. However, because the specimens were limited to renal tissue and peripheral blood, our study indicates only that NLRC5 may be associated with IgAN, and the role of NLRC5 in the mechanism of IgAN still needs further research. In addition, due to the limited observation time, our study lacks prognosis and survival data. Future studies should add follow-up monitoring while expanding on these data.

## Conclusions

Serum NLRC5 concentration is significantly decreased in IgAN patients, especially in group S1 of the Oxford classification, and it is negatively associated with the severity of renal pathology by Lee’s grade. In contrast, tissue NLRC5 concentration is significantly increased in IgAN renal specimens, especially in group S1 of the Oxford classification and is positively associated with the severity of disease by Lee’s grade. Measurement of NLRC5 may help with early diagnosis and monitoring of the progression of IgAN and could help avoid repeated renal biopsy, thus guiding accurate clinical decision-making and appropriate therapeutic interventions for IgAN patients. However, further mechanistic studies are needed to fully explore the roles of NLRC5 in IgAN.

## Abbreviations

NLRC5: nucleotide oligomerization domain-like receptor subfamily C5
IgAN: IgA nephritis
ELISA: enzyme-linked immunosorbent assay
NLRs: nucleotide binding oligomerization domain-like receptors
LRRs: leucine-rich repeats
PBS: phosphate buffer solution
AOD: average optical density
M ± SD: mean ± standard
ANOVA: one-way analysis of variance
M: mesangial cell proliferation score
S: segmental glomerulosclerosis or adhesion
E: intra capillary proliferative lesions
T: interstitial fibrosis/renal tubular atrophy
C: crescent
TGF-β1: transforming growth factor beta 1
AKI: acute renal failure
DN: diabetic nephropathy
